# The ultimate anticoagulants?

**Published:** 2010-02

## Introduction

Two new anticoagulants that can be administered orally are poised to change the face of anticoagulation as we know it. Rivaroxaban and dabigatran (neither available in South Africa as yet) have both shown positive results in clinical trials. At the congress of the South African Society of Thrombosis and Haemostasis, held in Johannesburg on 22 November 2009, Profs Sylvia Haas and Ajay Kakkar presented the evidence for rivaroxaban and dabigatran, respectively.

While very positive about the promise of rivaroxaban, Prof Haas of Technical University, Munich, Germany, cautioned nonetheless that ‘what is ultimate today may not be ultimate tomorrow’, given a constantly changing environment. ‘Rivaroxaban is a factor Xa inhibitor, and this factor is an attractive target for anticoagulation, as it prevents thrombin generation without blocking various other effects. Targeting it is therefore a very efficient mechanism of inhibition of thrombin generation. When factor Xa is activated, there is an immediate downward shift in thrombin concentration. Inhibiting it is therefore a sensible strategy for preventing a thrombin burst.’

Rivaroxaban is a once-daily, selective, oral, direct factor Xa inhibitor with potential value in a broad variety of clinical indications. Phase three studies for the primary prevention of venous thrombo-embolism (VTE) are already complete. Prof Haas reviewed the findings of the RECORD programme, which evaluated rivaroxaban relative to parenteral enoxaparin, a low-molecular weight heparin, in over 12 000 patients. The programme comprised four studies in total, allowing for meta-analysis. Two studies (RECORD 1 and 2)^1^,^2^ compared the use of the drugs following hip-replacement surgery and two (RECORD 3 and 4)^3^,^4^ compared them in knee-replacement surgery.

‘RECORD 1, designed as a noninferiority trial, showed the clear-cut superiority of rivaroxaban. RECORD 2, designed as a superiority trial, confirmed the safety and efficacy of rivaroxaban given as long-term prophylaxis to prevent VTE after total hip replacement. There were no other types of major bleeding associated with its use. The findings also showed that extended prophylaxis was superior to that of short duration. Where this medication is not yet included in anticoagulation guidelines after major orthopaedic surgery, we need to motivate for the necessary amendments’, Prof Haas commented.

RECORD 3 and 4 showed similar patterns in respect of total knee replacement. Once again, there was clear-cut superiority of rivaroxaban with regard to preventing symptomatic VTE and reducing all-cause mortality. It was non-inferior to enoxaparin when it came to clinically relevant non-major bleeding and cardiovascular events. ‘In summary, the RECORD programme showed that rivaroxaban significantly reduced VTE events without increasing bleeding rates.’

Various other phase III trials, such as MAGELLAN and EINSTEIN, are evaluating rivaroxaban in other settings, including deep-vein thrombosis and pulmonary embolism, and are still ongoing. ‘If the results are good, they will establish rivaroxaban as a new concept of VTE therapy – a single anticoagulant that replaces two different anticoagulants used for initial and acute therapy as well as for long-term anticoagulation’, observed Prof Haas.

The ROCKET study is comparing a fixed dose of rivaroxaban in atrial fibrillation and evaluating stroke prevention compared with standard warfarin treatment. Results are expected in 2010. ATLAS TIMI 5, evaluating rivaroxaban’s role in acute coronary syndrome, is expected to deliver results in either 2010 or 2011.

Prof Haas summarised the clinical utility of rivaroxaban as follows:

● it is formulated as a small tablet to be taken once daily
● the first pill is given six to eight hours postoperatively
● there is no need for injections or routine anticoagulation monitoring
● there are no problems associated with switching from one compound to another
● it can be combined with aspirin and NSAIDs (non-steroidal anti-inflamatories)
● it has potent, rapid anticoagulant effects (within two to four hours)
● it has high oral bioavailability (> 80%)
● it has low potential for food–drug and drug–drug interactions
● It can be given in a fixed dose, regardless of age, gender or extreme body weight.

‘And there is no toll to pay in respect of liver toxicity, renal effects and other side effects generally’, she concluded. Prof Ajay Kakkar, of the Thrombosis

Research Institute, Barts, and the London School of Medicine and Dentistry, made the case for dabigatran being the ‘ideal anticoagulant’ as he reviewed the data on its efficacy in preventing and treating VTE.

In his view, the ideal anticoagulant should be a once-daily treatment that can be given in a fixed dose, in or out of hospital. It needs to be predictable with a wide therapeutic window. It should require no monitoring and have minimal interactions with food or other drugs.

‘Thrombin is the ultimate part of the coagulation cascade. It therefore makes sense to target it. Like rivaroxaban, dabigatran is an orally administered medication. Unlike rivaroxaban, it targets thrombin (activated Factor II) and is a pro-drug that binds quickly to the thrombin burst with high affinity and specificity. It is predictable and is given in a fixed dose with no need for monitoring.’

The RE-NOVATE trial5 evaluated dabigatran (220 or 150 mg) versus enoxaparin (40 mg) in total hip replacement, while RE-MODEL^6^ and RE-MOBILIZE7 looked at the drug in total knee replacement. When the VTE data were viewed in their totality, there was a non-inferiority of dabigatran to enoxaparin in RE-NOVATE versus RE-MODEL but against the higher dose of enoxaparin 60 mg in RE-MOBILIZE, dabigatran failed to achieve non-inferiority. (Prof Kakkar pointed out that the higher rates of bleeding seen in these studies needed to be viewed in the context of the trial programme, including the actual surgical site in its evaluation.) The findings on major VTE versus total VTE mirrored each other, with both doses of dabigatran showing non-inferiority visa- vis enoxaparin.^8^

There are two distinct phases to consider in the treatment of VTE9 – managing acute events and preventing secondary events. The RE-COVER study of acute symptomatic VTE, the first of four treatment studies, randomised 2 500 patients to standard care with warfarin or to dabigatran. The question this study addressed was: ‘Can we replace the warfarin element with dabigatran while maintaining the initial parenteral phase with a low-molecular weight heparin’, Prof Kakkar noted.^10^

‘The results showed highly significant non-inferiority of dabigatran in the treatment of VTE after initial use of low-molecular weight heparin. Major bleeding was not significantly lower with dabigatran and all bleeding wassignificantly lower. This means that beyond the benefits we’ve seen with prophylaxis, dabigatran also has the potential to replace warfarin in the treatment of VTE. Warfarin’s narrow therapeutic window has always been problematic, in that if you fall outside it, you disadvantage the patient.’

A long-acting agent like dabigatran also offers the potential for a better option for stroke prevention. This strong rationale for using a thrombin inhibitor for stroke prevention underpinned the RE-LY study, which looked at patients with atrial fibrillation plus one stroke risk factor. Two doses of dabigatran (150 and 110 mg) were evaluated relative to warfarin.

‘RE-LY delivered striking results’, Prof Kakkar continued. ‘It was designed as a non-inferiority trial and while the lower dose of dabigatran did indeed emerge as non-inferior to warfarin when it came to preventing stroke, the higher dose was found to be superior to warfarin for stroke prevention. In addition, although one would intuitively expect a novel anticoagulant to cause more bleeding than warfarin, it turned out that there was no difference in bleeding rates for warfarin and the higher dose of dabigatran, while the lower dose was associated with a lower incidence of bleeding.’ He added the qualifier that while there was less major bleeding overall with dabigatran, the higher dose was, however, associated with increased gastrointestinal bleeding specifically.

Dabigatran also had a striking impact on haemorrhagic stroke. Both doses were associated with a lower incidence of intracranial haemorrhage. ‘This is a very positive finding for the oral agent, suggesting we can overcome the risk of intracranial haemorrhage associated with warfarin’, noted Prof Kakkar. Balancing this, however, was a higher risk of myocardial infarction with dabigatran, which he described as ‘difficult to understand’. Dabigatran also carried a higher risk for dyspepsia. ‘However, in terms of net clinical benefit, the lower dose of dabigatran was equal to warfarin and the higher dose was superior. Warfarin used to be our gold standard – but the thrombin inhibitor appears to be an improvement.’

Turning to acute coronary syndrome, Prof Kakkar observed, ‘we need to do better in reducing recurrent events (stroke, myocardial infarction, angina) at day 30. Currently there are only phase II data on dabigatran in this context, but they validate that targeting a thrombin inhibitor might work.’

Summing up, Prof Kakkar stated that extensive clinical evaluation has validated the benefits of thrombin inhibition as well as its safety. ‘It may be too early to call dabigatran “the ultimate coagulant”, but there’s no question that it will be very useful to us. In addition, all the ongoing clinical trials will provide us with study subjects we didn’t have before, allowing us to answer various questions that will ultimately improve clinical outcomes for our patients.’

## Stop Press

Xarelto 10® has now been registered in South Africa. Xarelto 10 film-coated tablets are indicated for the prevention of VTE in patients undergoing major orthopaedic surgery of the lower limbs. For more information contact Sandhya Misra at Bayer Schering Pharma on 011 921-5021.
